# Effect of a novel functional tomato sauce (OsteoCol) from vine-ripened tomatoes on serum lipids in individuals with common hypercholesterolemia: tomato sauce and hypercholesterolemia

**DOI:** 10.1186/s12967-020-02676-3

**Published:** 2021-01-06

**Authors:** Yvelise Ferro, Elisa Mazza, Elvira Angotti, Roberta Pujia, Angela Mirarchi, Maria Antonietta Salvati, Rosa Terracciano, Rocco Savino, Stefano Romeo, Antonio Scuteri, Rosario Mare, Francesco Saverio Costanzo, Arturo Pujia, Tiziana Montalcini

**Affiliations:** 1 Department of Health Science, University Magna Grecia, 88100 Catanzaro, Italy; 2 Department of Medical and Surgical Science, Nutrition Unit, University Magna Grecia, 88100 Catanzaro, Italy; 3 Department of Clinical and Experimental Medicine, Nutrition Unit, University Magna Grecia, 88100 Catanzaro, Italy; 4 Department of Molecular and Clinical Medicine, University of Gothenburg, 41101 Gothenburg, Sweden; 5 Primary Care Unit of Borgia, 88021 Catanzaro, Italy

**Keywords:** Functional food, Tomato sauce, Sterols, Carotenoids, Lipids, Cholesterol

## Abstract

**Background:**

Most studies focused on the benefits of lycopene on serum lipids but no studies have been specifically designed to assess the role of a tomato sauce from vine-ripened tomatoes on patients affected by polygenic hypercholesterolemia. The aim of this study was to compare the lipid-lowering effect of a novel functional tomato sauce with a well-known functional food with a lipid-lowering effect, i.e. a sterol-enriched yogurt.

**Methods:**

In this cross-over study, we evaluated a population of 108 ambulatory patients affected by polygenic hypercholesterolemia of both gender, who were allocated to a tomato sauce (namely OsteoCol) 150 ml/day or a sterol-enriched yogurt (containing sterols 1.6 g/die) treatment, for 6 weeks. Carotenoids content was 3.5 mg per gram of product. We measured serum lipids and creatinine and transaminases at basal and follow-up visit.

**Results:**

A total of 91 subjects completed the protocol. A significant difference in LDL-cholesterol change was found between participants taking yogurt, tomato sauce (high adherence) and tomato sauce (low adherence) (− 16; − 12*;* + 8 mg/dl respectively; p < 0.001). We found a greater LDL-cholesterol reduction in the participants with a basal LDL-cholesterol more than 152 mg/dl (15% for sterol-enriched yogurt and 12% for tomato sauce at high adherence).

**Conclusion:**

A novel functional tomato sauce from vine-ripened tomatoes compares favourably with a commercialised sterol-enriched yogurt in term of absolute LDL-cholesterol change. Intake of a tomato sauce with a high carotenoid content may support treatment of patients affected by common hypercholesterolemia. The present study has various limitations. The presence of other dietary components, which may have influenced the results, cannot be ruled out. Of course, these results cannot be extrapolated to other populations. Furthermore, there was a low adherence rate in the tomato sauce group. Moreover, we did not report serum carotenoids data.

*Trial registration*: ID: 13244115 on the ISRCTN registry, retrospectively registered in 2019-5-14. URL: http://www.isrctn.com/ISRCTN13244115

## Background

Carotenoids are a class of more than 700 naturally occurring pigments synthesized by plants [1]. Dietary carotenoid intake as well as the intake of specific carotenoids (such as α- and β-carotene, β-cryptoxanthin and lycopene) have been inversely associated with coronary heart disease, stroke, and mortality [2]. In addition, blood carotenoid concentrations have been inversely associated with cardiovascular disease, total cancer, and all-cause mortality [2].

It has been suggested that it would be reductive to explain the physiological effects of carotenoids solely by their antioxidant activity [3]. In fact, it has also been demonstrated that β-carotene regulates the expression of the HMG-CoA reductase enzyme in rat liver [4], both β-carotene and lycopene have been reported to inhibit macrophage HMG-CoA reductase activity [5] and fucoxanthin, a marine carotenoid, modulates both the HMG-CoA reductase and acyl-coenzyme A [3], all resulting in the inhibition of cholesterol synthesis.

It has been found that at least 25 mg per day of lycopene (obtained through both diet and supplementation) elicit beneficial health effects, helping lower total cholesterol levels by an average of 8 mg/dl [6]. A study showed an average 18 mg/dl (9%) decrease with up to 35 mg per day [7]. In another study, consumption of astaxanthin 6 and 12 mg/day significantly increased serum high-density lipoprotein cholesterol (HDL-C) versus baseline, and doses of 12 and 18 mg/day significantly decreased serum triglyceride levels [8].

An intake of 300–400 g/day of vegetables provides at least 25 mg of total carotenoids/day [9]. However, since tomato (*Solanum lycopersicum* L.) is a fruits rich in various carotenoid pigments, especially lycopene, and it is among the most widely consumed crops, it represents the most important source of these molecules for human health. Tomato contains a complex mixture of carotenoids, including lycopene (35–96% total lycopene, primarily in all *trans*-isomeric forms, and 1–22% *cis*-lycopene), β-carotene and lutein, which all support cardiovascular health. The regular consumption of tomato and tomato-based products have been correlated to a reduction in risk of contracting cardiovascular diseases confers cardiovascular benefits [10]. Several studies support the notion that the intake of tomato-based foods improves serum lipids and reduces the cardiovascular risk better than lycopene supplementation [11–13].

In this regard, it has also been demonstrated that lycopene or lycopene-containing products are effective in lowering systolic blood pressure, in particular in hypertensive subjects and at high dosage (> 12 mg/day) [3, 14].

However, carotenoids content varies significantly between cultivars, growing conditions as well as stage of maturity and storage temperatures [15–17]. Tomatoes picked green and ripened in storage usually have lower levels of carotenoids than vine-ripened fruit. This is a common commercial practice, although the quality of tomatoes ripened on-the-vine may be better than tomatoes ripened off-the-vine. Several studies have confirmed that vine-ripened tomatoes maintain the phytochemical content better than tomatoes ripened off the plant [18–20]. Currently there is a lack of information about the influence of tomato ripeness stage on the carotenoids content. Thus, specific evaluations of the tomato carotenoids content and the cardiovascular benefits are required before conferring a nutraceutical/therapeutic value to a tomato-based food.

Long-term adherence to diet and lipid-lowering agents is a key issue. Functional foods and nutraceuticals can be natural alternatives and support to pharmacological therapies in statin-intolerant patients, because they might significantly reduce LDL-C [21]. Moreover, functional foods exert other non–lipid-lowering properties, including reduction of glucose, blood pressure and inflammation, and treatment with functional foods seems to be very safe and well tolerated.

Our study investigated the effects of a tomato sauce from vine-ripened tomatoes on lipids in individuals affected by common hypercholesterolemia. We compared the lipid-lowering effect of this functional tomato sauce with a well-known commercialized functional food with a lipid-lowering effect, i.e. a sterol-enriched yogurt.

## Methods

### Subjects

A population of one hundred and eight subjects (40 male and 68 female), with newly diagnosed hypercholesterolemia, not taking lipid-lowering medication, nutraceuticals, supplements or functional food, and aged between 20–75 attending the outpatient lipid clinic of the “Mater Domini” Azienda University Hospital in Catanzaro, Italy, were enrolled for this study (enrolment period between February 12, 2018 and July 19, 2018). The study’s protocol allowed to enroll individuals who had taken lipid-lowering drugs or other up to three months before but we chose to enroll only those who had never used these medications.

All of them were affected by polygenic hypercholesterolemia, that is the most common primary disorder causing an increase in plasma LDL-C associated with a low-moderate risk for coronary artery disease and causing hyperlipidemia only in 10% of the first-degree relatives [22]. We excluded individuals with familial hypercholesterolemia causing an increase in total cholesterol concentrations above the 95th percentile, hypercholesterolemia in 50% of the first-degree relatives, the presence of tendon xanthomata and a history of early coronary heart disease [i.e. before age 55 in men or 65 in women) [22].

Furthermore, according to the protocol of the study, we excluded subjects with triglycerides concentration over 150 mg/dl, and those with nephrotic syndrome, chronic renal failure, and allergies to milk proteins, soy and tomato and those suffering from gastrointestinal diseases or who had cardiovascular events in the previous 6 months. In addition, although not specified in the protocol and according to other studies on this issue we excluded those with secondary causes of hyperlipidemia [as cholestasis, hypothyroidism, pregnancy, sepsis, acute intermittent porphyria, oral contraceptive use, corticosteroid therapy, immunosuppression, past and current alcohol abuse (> 20 g of alcohol per day; 350 mL (12 oz) of beer, 120 mL (4 oz) of wine, and 45 mL (1.5 oz) of hard liquor each contain 10 g of alcohol], or who was affected by debilitating diseases, as ascertained from their clinical records.

### Study design

A cross-over study design was used. We performed a study lasting 6 weeks with the LDL-C as the main outcome. Ninety-one subjects completed the treatments that were as follows:

- 1. A functional tomato sauce [namely OsteoCol (registered Patent), from tomatoes ripened on-the-vine], 150 ml/day (provided by C.G. Food, SRL, Soverato, Italy);
- 2. A sterol-enriched yogurt (containing sterols 1.6 g/100 g/ day, provided by Danone, SPA, Milano, Italy).

In this type of study, the participants cross over from one arm of the study to the other and serve as their own control group.

Due to the different packaging of the treatments, in this study both the experimenters and participants were aware of who was receiving the tomato sauce or the yogurt, while data collectors and outcome adjudicators were blind. Furthermore, due to the expiry of the supply of yogurt in a very restricted period, we used a block randomization. However, to eliminate selection bias, we randomized patients in blocks at recruitment rather than as they arrived. Furthermore, to help to minimize potential bias, we assessed the baseline clinical characteristics of participants according to allocation.

Participants received oral and written recommendations to adhere to a Mediterranean dietary pattern, without energy restriction by dietitians [23] who also delivered the intervention. Both groups were under close dietetic supervision by a registered dietician through the entire study.

Macronutrient distribution as a percentage of total energy range from 50 to 55% carbohydrate, 15–20% protein and 20–35% fat, with a recommended protein intake of 1 g/kg of ideal body weight [23]. Contrary to the protocol, the yogurt was delivery at the clinic and not at home.

Local ethical committee at the “Mater Domini” Azienda University Hospital approved the protocol (06/2018/CE approved 18 January, 2018). Written informed consent was obtained from all participants. The investigation conforms to the principles outlined in the Declaration of Helsinki (The study is listed on the ISRCTN registry with study ID ISRCTN13244115).

**Fig. 1:**
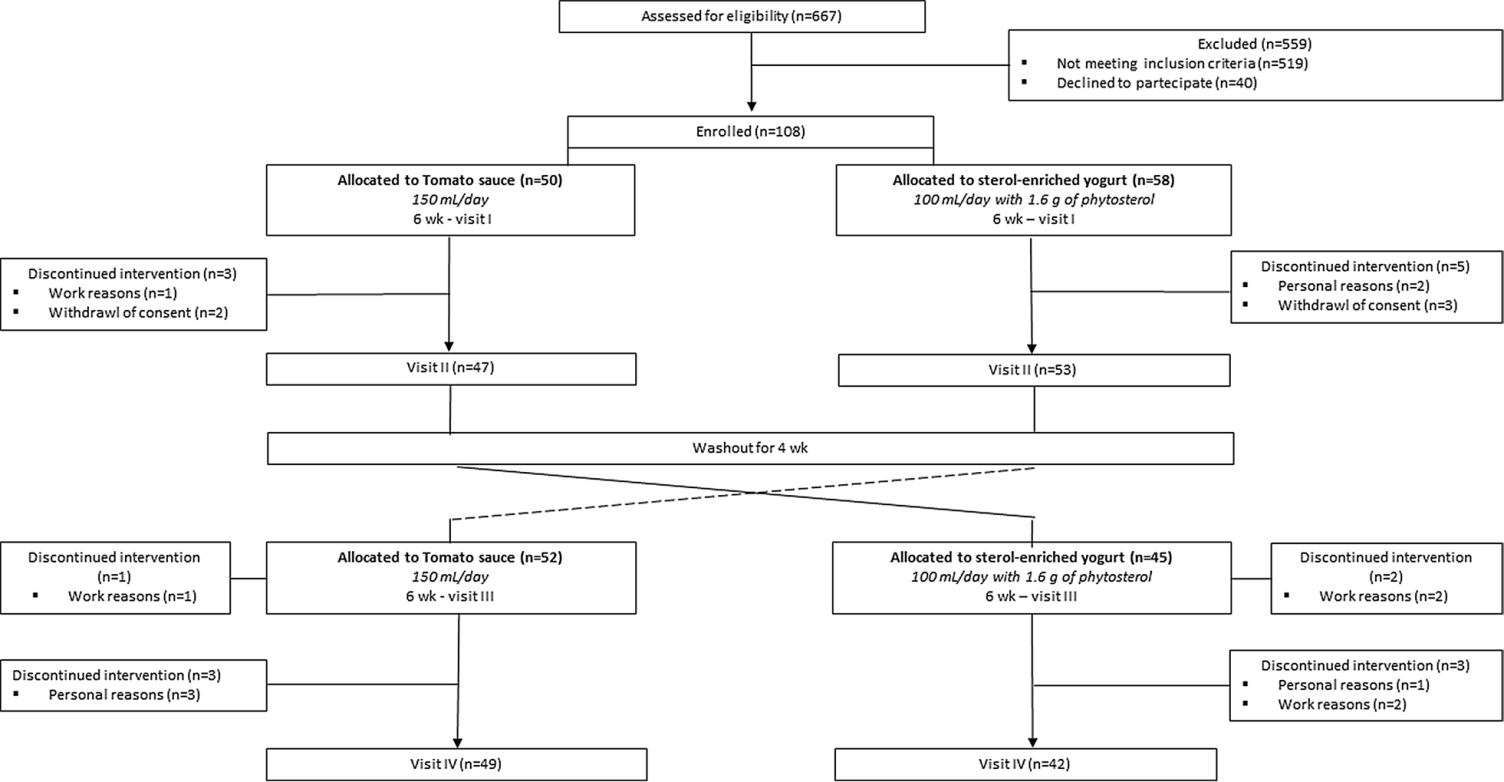
Flow-chart of the study

Figure 1 shows the flow-chart of the study (Last follow-up visit was in September, 1, 2018). Participants were advised to avoid consuming other tomato products throughout the study period. Subjects consumed their regular diet during the 4 weeks washout periods.

### Carotenoids content analysis

Carotenoid concentration in the tomato sauce was assessed by matrix-assisted laser desorption/ionizationTime of Flight (MALDI-TOF) mass spectometry (ABSciex, Framingham, MA, USA) [24]. Carotenoids content was 3.5 mg/g.

### Anthropometric measurements and cardiovascular risk factors assessment

Body weight was measured before breakfast after a 12 h overnight fast with the subjects lightly dressed, subtracting the weight of clothes. Body weight was measured on a calibrated digital scale (model Tanita BC-418MA) accurate to 0.1 kg, and standing height was measured with a wall-mounted stadiometer [23]. BMI was calculated with the following equation: weight (kg)/height (m)^2^. Waist circumferences and hip circumferences (WC and HC) were measured with a no stretchable tape over the unclothed abdomen at the narrowest point between costal margin and iliac crest and over light clothing at the level of the widest diameter around the buttocks, respectively, as described in the past [23].

We assessed the presence of the classical cardiovascular (CV) risk factors, such as hypertension, diabetes and smoking, from clinical records and patient interview [23, 25].

Blood pressure was determined at the time of the two visits, as previously described [23]

### Biochemical evaluation

Venous blood was collected after fasting overnight into vacutainer tubes (Becton & Dickinson, Plymouth, England) and centrifuged within 4 h. Serum glucose, total cholesterol, high density lipoprotein (HDL)-cholesterol, triglycerides, creatinine, high sensitivity C-reactive protein (CRP) and transaminases were measured by chemiluminescent immunoassay on COBAS 8000 (Roche, Switzerland), according to the manufacturer’s instructions. LDL- C level was calculated by the Friedewald formula [26]. Lipoprotein (a) was assessed with immunoturbidimetry method (normal value of < 75 nmol/L). Quality control was assessed daily for all determinations.

Three informed, consenting, healthy volunteers between the ages of 30 and 45 years and BMIs between 19–22 kg/m^2^ were recruited for the evaluation of serum lycopene concentration. Subjects were non-pregnant, non-smoking adults who were free from metabolic diseases. All subjects were instructed to not consume lycopene-containing foods for 7 days prior to serum lycopene evaluation and during the 4 experimental days. Lycopene concentration was assessed at the baseline and after four days of the consumption of 150 ml/day of the tomatoes sauce ripened on-the vine. Lycopene was quantified by HPLC method (Jasco LC-NET II).

### Data analysis

Data are reported as mean ± standard deviation (SD). Based on the assumption that the within-patient standard deviation of the response variable is 20, considering a probability of 95% that the study will detect a treatment difference at a two-sided 0.05 significance level, if the functional tomato sauce is not inferior to the sterol-enriched yogurt and the true difference between treatments is inferior to 10 mg, a total of 106 patients entered this two-treatment crossover study [27] (Fig. 1). Changes in the clinical characteristics from baseline to follow-up (within group variation) were calculated using paired Student’s *t* test (two tailed). ANOVA was used to compare the mean changes between LDL-C tertiles. The General Linear Model (GLM) was used to adjust the LDL-C reduction for potential confounders (such as weight change).

We used both an indirect assessment method (i.e., bottle caps counts) and patients interview for assessing adherence. We defined a participant as low adherent (LA) when the participant took less than 80% of the prescribed treatment.

Significant differences were assumed to be present at *p* < 0.05 (two-tailed). All comparisons were performed using SPSS 22.0 for Windows (IBM Corporation, New York, NY, United States).

## Results

Table 1 shows the basal clinical characteristics of participants who completed the study (n = 91). The mean age of the enrolled population was 54 ± 10 years. The mean basal LDL-C was 154 ± 18 mg/dl. A total of 40 (37%) were male, and 26% had hypertension. The prevalence remained stable during the entire study (also medications). In this study the drop-out rate was 15% and low-adherence rate was 33% (only in the tomato sauce group).

**Table 1:** Baseline demographic and clinical characteristics of participants who completed the study

Variables		Polygenic hypercholesterolemic patients (n = 91)
Age (years)		54 ± 11
Weight (kg)		68 ± 11
BMI (kg/m^2^)		2 ± 3
WC (cm)		93 ± 9
HC (cm)		102 ± 6
SBP (mmHg)		120 ± 13
DBP (mmHg)		75 ± 8
Glucose (mg/dL)		91 ± 11
Creatinine (mg/dL)		0.8 ± 0.2
TC (mg/dL)		236 ± 20
HDL-C (mg/dL)		62 ± 13
LDL-C (mg/dL)		154 ± 18
TG (mg/dL)		102 ± 29
Non HDL-C (mg/dL)		174 ± 20
AST (IU/L)		20 ± 6
ALT (IU/L)		20 ± 10
*Prevalence*		
Male % (n)		37 (34)
Menopause status % (n)		79 (45)
Smokers % (n)		34 (31)
Hypertension % (n)		26 (24)
Antihypertensive agents % (n)		26 (24)
Antiplatelet drug % (n)		7 (6)

*BMI* body mass index, *WC* waist circumference, *HC* hip circumference, *SBP* systolic blood pressure, *DBP* diastolic blood pressure, *TC* total cholesterol, *HDL-C* high density lipoprotein cholesterol, *LDL-C* low density lipoprotein cholesterol, *TG* triglycerides, *AST* aspartate aminotransferase, *ALT* alanine aminotransferase

In healthy volunteers, after four days of tomato sauce consumption, serum lycopene (all trans isomer) was 1.26 ± 0.04 µM/L while it was undetectable at baseline.

Changes in the clinical parameters after each treatment period are shown in Table 2. Both the sterol-enriched yogurt and Tomato sauce (high adherence- HA) significantly lowered serum LDL-C while the Tomato sauce-LA increased LDL-C (absolute difference: − 16 ± 21; − 12 ± 17*;* + 8 ± 15 mg/dl in the yogurt, Tomato sauce-HA and Tomato sauce-LA group, respectively; p < 0.001; p < 0.001 and p = 0.008 respectively; paired t-test).

**Table 2:** Baseline and follow-up clinical characteristics of participants according to the intervention and adherence group

Variables	Sterol-enriched yogurt (n = 91)				Tomato sauce HA (n = 61)				Tomato sauce LA (n = 30)			
	Before	After	Δ	*p-value*	Before	After	Δ	*p-value*	Before	After	Δ	*p-value*
Weight (kg)	67 ± 11	66 ± 11	− 1.2 ± 1	< 0.001	67 ± 10	66 ± 10	− 0.8 ± 1	< 0.001	67 ± 11	66 ± 11	− 1.1 ± 1	< 0.001
BMI (kg/m^2^)	25 ± 3	25 ± 3	− 0.4 ± 0.5	< 0.001	25 ± 3	25 ± 3	− 0.3 ± 0.6	< 0.001	25 ± 3	25 ± 2	− 0.4 ± 0.5	< 0.001
WC (cm)	91 ± 9	90 ± 9	− 1.5 ± 3	< 0.001	90 ± 9	89 ± 8	− 0.9 ± 3	0.05	90 ± 9	88 ± 8	− 2.4 ± 4	0.008
HC (cm)	100 ± 6	99 ± 6	− 1.5 ± 2	< 0.001	100 ± 7	99 ± 6	− 1.5 ± 2	< 0.001	99 ± 5	98 ± 6	− 0.8 ± 2	0.09
SBP (mmHg)	119 ± 13	117 ± 11	− 2 ± 10	0.06	119 ± 13	115 ± 12	− 3 ± 12	0.040	117 ± 11	116 ± 13	− 0.7 ± 11	0.74
DBP (mmHg)	74 ± 7	74 ± 6	− 0.01 ± 8	0.98	75 ± 8	72 ± 7	− 3 ± 8	0.010	74 ± 6	74 ± 8	0.2 ± 9	0.89
Glucose (mg/dL)	89 ± 11	89 ± 13	0.2 ± 10	0.87	91 ± 9	89 ± 9	− 2 ± 8	0.019	88 ± 8	84 ± 6	− 3 ± 7	0.010
Creatinine (mg/dL)	0.80 ± 0.2	0.83 ± 0.2	0.03 ± 0.1	0.003	0.80 ± 0.1	0.79 ± 0.1	− 0.01 ± 0.1	0.13	0.83 ± 0.2	0.80 ± 0.1	− 0.03 ± 0.1	0.035
TC (mg/dL)	230 ± 26	211 ± 26	− 18 ± 24	< 0.001	227 ± 25	216 ± 24	− 11 ± 18	< 0.001	220 ± 23	230 ± 27	10 ± 17	0.003
HDL-C (mg/dL)	60 ± 12	58 ± 12	− 3 ± 6	< 0.001	60 ± 14	59 ± 12	− 0.8 ± 6	0.031	56 ± 13	56 ± 12	0.1 ± 6	0.95
LDL-C (mg/dL)	148 ± 24	132 ± 23	− 16 ± 21	< 0.001	147 ± 20	135 ± 18	− 12 ± 17	< 0.001	142 ± 19	150 ± 23	8 ± 15	0.008
TG (mg/dL)	106 ± 38	106 ± 46	0.5 ± 40	0.91	100 ± 34	109 ± 44	9.0 ± 27	0.012	105 ± 32	117 ± 49	12 ± 31	0.049
Non HDL-C (mg/dL)	169 ± 26	154 ± 25	− 16 ± 22	< 0.001	167 ± 23	158 ± 22	− 9 ± 15	< 0.001	163 ± 21	174 ± 27	10 ± 16	0.002
AST (IU/L)	19 ± 5	19 ± 5	0.1 ± 4	0.86	19 ± 5	20 ± 5	0.4 ± 4	0.38	19 ± 3	19 ± 4	0.01 ± 3	0.94
ALT (IU/L)	19 ± 9	18 ± 6	− 0.5 ± 8	0.50	18 ± 7	19 ± 7	0.5 ± 4	0.32	17 ± 8	17 ± 7	− 0.8 ± 6	0.43

Δ, changes; BMI, body mass index; WC,  waist circumference; HC, hip circumference; TC, total cholesterol; HDL-C, high density lipoprotein cholesterol; LDL-C, low density lipoprotein cholesterol; TG, triglycerides; AST, aspartate aminotransferase; ALT, alanine aminotransferase

Body weight, WC, HC, TC and non HDL-C significantly reduced after each treatment (for body weight: − 1.2 ± 1; − 0.8 ± 1; − 1.1 ± 1, in the yogurt, Tomato sauce-HA and Tomato sauce-LA group, respectively; p < 0.001 for all; Table 2). SBP and DBP significantly reduced only after the Tomato sauce (HA) (− 3 ± 12 and − 3 ± 8 respectively; p = 0.04 and p = 0.001, Table 2). Furthermore, creatinine increased and HDL-C decreased significantly after the sterol-enriched yogurt while TG increased and HDL-C and glucose decreased after the Tomato sauce-HA and TG increased and glucose decreased after the Tomato sauce-LA. No other variables significantly changed at follow-up visit.

**Fig. 2:**
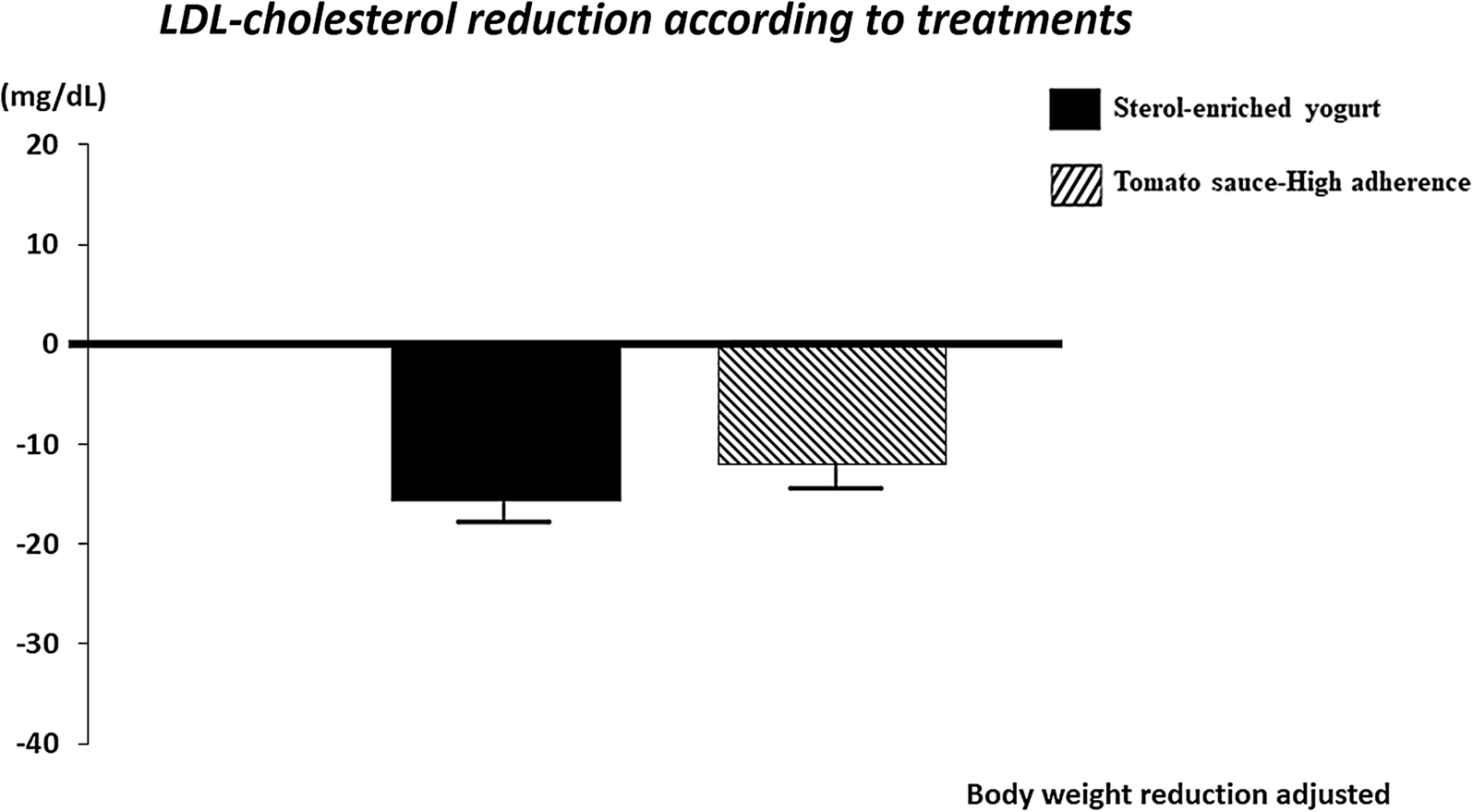
LDL-C reduction according to the treatment

LDL-C reduction, which was adjusted for body weight change, was − 15.3 ± 2 and − 12.4 ± 2 mg/dl in the yogurt and tomato sauce-HA, respectively (p = 0.35, see Fig. 2);

The servings of various food categories consumed daily or weekly during the study is presented in Additional file 1: Table S1. Dietary intake was not significantly different between groups except for a sterol-enriched yogurt (1 serving/day in the yogurt group) and Osteocol consumption (1 serving/day) in the Tomato sauce group.

Additional file 2: Figure S1 shows individual LDL-C change for the participant after each treatment. Overall, allocation to the Tomato-sauce resulted in a 10 mg/dl median reduction in LDLC. However, individual variability in per cent LDLC reduction was wide ranging from modest increases to reductions exceeding 50%.

**Fig. 3:**
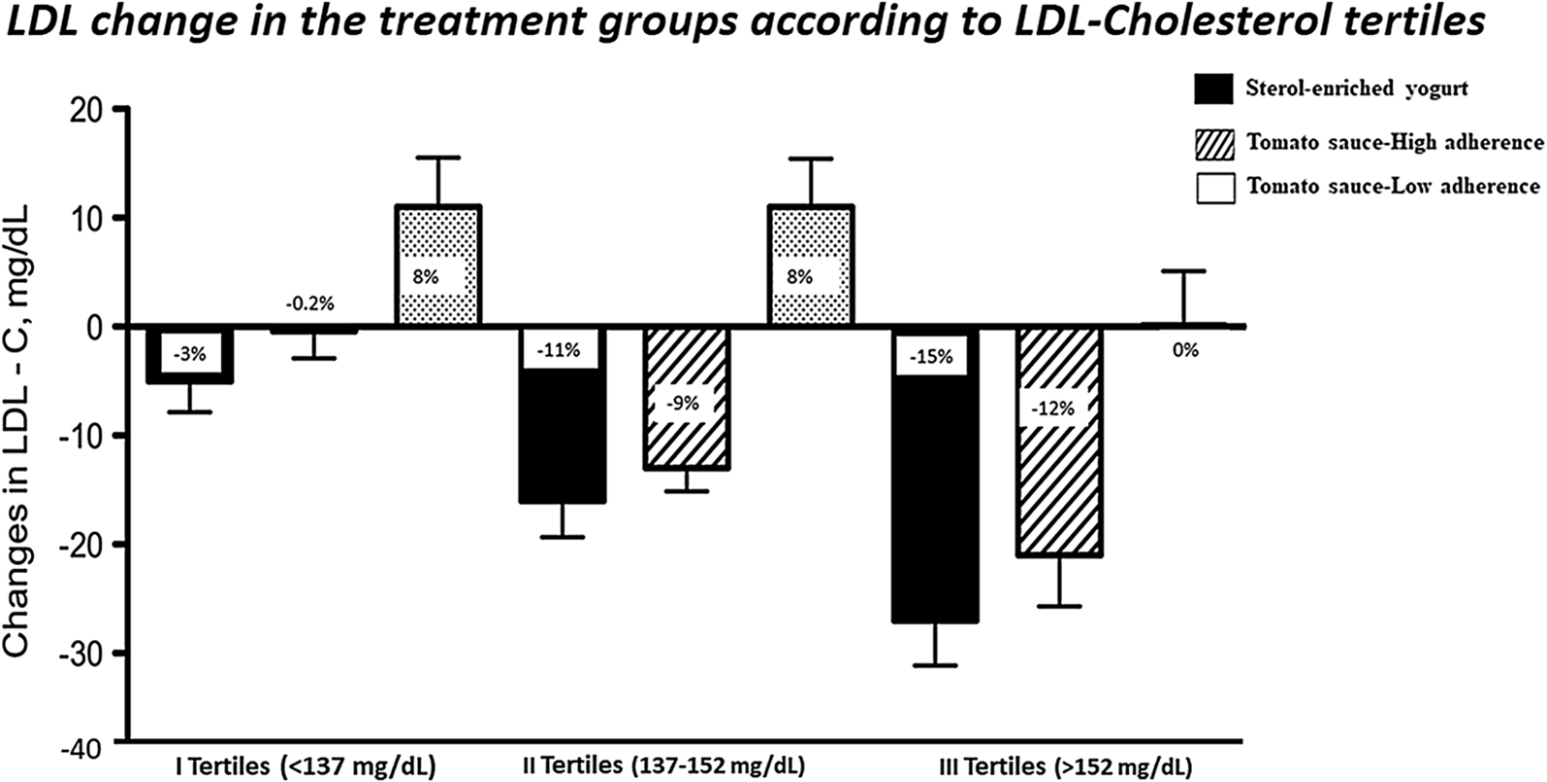
LDL-C change in the treatment group according to LDL-cholesterol tertiles

Figure 3 shows the population categorised according to the basal LDL-C tertiles. In the highest tertile (with LDL-C more than 152 mg/dl) the average LDL-C decrease was − 15% for sterol-enriched yogurt and − 12% for Tomato sauce (HA).

Additional file 3: Figure S2 shows the mean high sensitive CRP change (− 21%) as well as individual reduction after Tomato sauce intake. Individual variability in per cent CRP reduction was wide ranging, with reductions exceeding 30%.

Moreover, in those in the Tomato sauce-HA group there was a significant reduction of the Lp (a) concentration after 6 weeks (at Baseline: Lp (a) 34.5 ± 43 nmol/L; at 6 week: 29.4 ± 36 nmol/L, p = 0.019; Additional file 4: Figure S3).

All the participants had no adverse symptoms during the entire study period.

## Discussion

With a constant increase in the overall age of the population, chronic diseases of aging impose an enormous cost of health care. Preventative healthcare strategies, which include nutritional approaches, could save billion in annual healthcare costs. An increasing numbers of consumers are turning to their diet for well-being and consumer interest in self-care is a leading factor motivating functional foods consumption in place of several medications. Furthermore, due to statin-associated muscle symptoms, adherence to statin therapy for the reduction of LDL-C is challenging in clinical practice. Certain functional foods might be considered as an alternative or add-on therapy to statins. These products could also exert multiple non–lipid-lowering actions, including anti-inflammatory and antioxidative properties.

In the present clinical trial, we demonstrate that a novel functional tomato sauce (named OsteoCol) reduces LDL-C concentrations by 5–10% (− 7.6 to − 16.1 mg/dl) in the participants with a high adherence to the protocol (i.e.; > 80% of the prescribed treatment) after intervention for six weeks.

The change observed in LDL-C was in the range of those obtained with another recognized cholesterol-lowering functional food which was a sterol-enriched yogurt (− 6.6 to − 12.6%). Furthermore, we found a significant reduction in TC and non HDL-C.

It has been demonstrated, in postmenopausal women, that consuming at least seven servings/week of lycopene-based products decreases cardiovascular risk within 7 years [28]. Since a reduction of 10 mg/dl in LDL-C concentration has been associated with a significant reduction in the development of coronary heart disease (6.6%), major vascular events (5.8%), stroke (4.1%), and mortality (4.6%) [29], our results are of important clinical significance.

Previous studies have demonstrated a modest reduction of LDL-C (of ~ 5 mg/dl) with lycopene-containing foods or lycopene as a nutraceutical [6], while other investigations have found a 10% reduction (mean ΔLDL of ~ 10 mg/dl) [30–34], therefore we choose a nutraceuticals with proven efficacy as control.

We found a higher lipid-lowering effect in the participants of the highest basal LDL-C tertiles (− 15% with a sterol-enriched yogurt and − 12% with Tomato sauce). With a mean reductions of 12%, the cholesterol-lowering efficacy of the tomato sauce tested in the current trial compares favourably with the results of other functional foods or nutraceuticals containing oats-based fibers or glucomannan or catechins or chitosan as well as a low doses of statin drugs [35, 36].

According with the current dietary guidelines that are instruments of public health policy to promote a healthy diet [37] which suggest consuming a more than five cups/week of red vegetables, in our study the daily consumption of tomato sauce was 150 ml. Furthermore, this study is in line with recent guidelines [38] suggesting treating patients with mild-to-moderate hypercholesterolemia who already follow a healthy diet with cholesterol-lowering functional foods (or nutraceuticals) to reduce LDL-C levels.

Research into the treatment of hypercholesterolemia is progressive. Other than classical medical treatments, several forms of alternative therapies—such as functional foods, nutraceuticals and other dietary supplements—have also been tested for these patients.

The lipids reducing properties of tomato have been associated with the suppression of cholesterol synthesis via the inhibition of HMG-CoA-reductase and activation of LDL-receptors [39]. In line with this potential mechanism, we did not assessed any markers for cholesterol absorption.

Since a tomato sauce can be defined as a functional food only if, in addition to its nutritional properties, it has additional properties for health, in this original study we tested the lipid-lowering effects of a tomato sauce from vine-ripened tomatoes in individuals affected by common hypercholesterolemia. Todays, the vast majority of tomato crops are harvested at a mature green stage and are ripened off the plant with consequent lower levels of carotenoids and lycopene compared to those ripened on the vine [20]. Ripening‐induced changes in tomato matrix influences the amount and bioaccessible fraction of carotenoids in tomato‐based products. Using a spectrophotometric method, a study demonstrated that total carotenoids content markedly increased as fruits ripened, ranging from 0.53 ± 0.11 mg/kg at mature‐green stage to 14.82 ± 1.62 mg/kg at the most advanced stage of ripeness [40, 41]. Changes in lycopene content during tomato ripening showed a similar pattern [40]. It has been reported a maximum content of total carotenoids in tomato juice samples of 60 mg/kg and in dried tomato of 1.3 g/kg [42].

In our tomato sauce the carotenoids content was of 3.5 mg per gram of product, which is higher than other cultivar or commercial varieties [40–44]. Thus, ripening tomato on the vine could be an excellent strategy to positively influence the nutritional quality of tomato. Of course, the overall health benefits observed with the tomato sauce consumption could also be due to the combined effects of all the carotenoids.

Body weight significantly changed during the study but in a minimal extent (− 1.2 ± 1; − 0.8 ± 1; − 1.1 ± 1, in the yogurt, Tomato sauce-HA and Tomato sauce-LA group, respectively; Table 2). Our study was not designed to explore this specific aspect. When fucoxanthin, a marine carotenoid, at a dose of 24 mg/die was administered to obese women for 16 weeks, a significant reduction of body weight, WC and liver fat content, with a significant increase in resting energy expenditure, was found [45]. By modulating the adaptive thermogenesis, fucoxanthin plays a crucial role in energy expenditure [3]. Future studies could elucidate if carotenoids from vine-ripened tomato would have anti-obesity proprieties. Interventions aimed at weight loss reduce lipids in blood. However, weight loss did not explain LDL-C reduction in our population. In fact, the LDL-C reduction, which was adjusted for body weight change, was of the same entity in both the yogurt and tomato sauce-HA groups (− 15.3 ± 2 and − 12.4 ± 2 mg/dl, respectively; p = NS, Fig. 2);

The mechanisms underlying the reduction of LDL-C may or may not be related to carotenoids or lycopene and we cannot rule out the possible role of other nutrients. For example, sterols are one of the classes of components contained in tomato. Sterols are integral membrane components of the tomato and may have both a structural and a metabolic function [46]. Nevertheless, the content of phytosterols in dried tomato is less than 100 mg/kg [47]. It has been reported that the concentration of phytosterols in a vegetable juice is very low [48]. Only after consuming more than 400 tomatoes one gets more than 1 g of plant sterols, necessary to induce cholesterol lowering effect.

Thus, we did not assess phytosterols either in the tomato sauce or in the serum of the participants. Further studies are needed to better clarify the role of phytosterols in tomato-based products.

In this study we found that a yogurt containing 1.6 g of plant sterols reduced HDL-C (~ − 3 mg/dl; Table 2). This finding is in line with a previous study in which a yogurt providing 2 g per day of a plant stanol ester mixture, containing sitostanol and campestanol for five weeks, reduced HDL-C by 2.5% in hypercholesterolemic subjects [49]. However, HDL-C also reduced significantly in the tomato sauce –HA group (p = 0.003; Table 2). These results are similar to those of other intervention studies in which HDL-C reduced at the end of the study, independent of the treatment [50–52]. At this moment, the underline mechanism is unclear. Despite significant from a statistical point of view, the clinical implications of changes in HDL-C (− 3 mg/dl), creatinine (+ 0.03), glucose (− 2 mg/dl), triglycerides (+ 9 mg/dl; Table 2), may be very modest or absent and a longer study is needed to confirm these findings and better compare the two functional foods tested in the current trial. In the case of triglycerides it is possible that the cultivation technique increases the tomato fructose content [53]; in some short-term controlled feeding studies, dietary fructose significantly increased TG levels [54]. However, this effect is overwhelmed by the significative effect on Non-HDL cholesterol (Table 2).

With this study, it was not our intention to investigate the efficacy of the sterols-enriched yogurt, which was already well-established [55]. Since the health claims relating to the cholesterol-lowering effect for tomato sauces have not yet been approved by EFSA, we thought that the best reference for our comparison could be a sterols-enriched yogurt, for which health claims are approved, but not a standard tomato sauce.

Furthermore, we have chosen a sterols-enriched yogurt due to the fixed sterols dose, as well as for its wide commercialization and acceptability. The use of other functional foods, such as oats or barley, make less feasible to assess the treatments adherence. Most important, both the yogurt and the tomato sauce are consumed daily. The tomato sauce is consumed daily in the Italian Mediterranean cuisine. For all these reasons, we compared the tomato sauce to a sterol-enriched yogurt.

In an our previous investigations, no significant variations in plasma lathosterol, campesterol or s-sitosterol concentrations after 6 weeks of treatment with a sterol-enriched yogurt was found [56].

Thus, considering the aim of the study and the considerable amount of heterogeneity in plasma phytosterols concentrations amongst the studies [55], we did not assess serum phytosterols. Moreover, in this study, all participants took the yogurt daily as planned.

Although there are no RCTs showing the effects of long-term plant sterol intake on cardiovascular disease (CVD) outcomes, e.g., CV events [57], it seems reasonable that sterol consumption may lower CVD risk based on the established LDL-C lowering effect. Furthermore, the demonstration of an association between moderately increased plant sterol plasma levels and low risk for coronary heart disease (CHD) may contributes to the understanding of the significance of dietary plant sterols for the human health. The EPIC Spanish cohort [58] observed that elevated levels of plasma sitosterol, the main dietary phytosterol, was inversely related to CHD. The results of a community-based study on elderly subjects showed that high plant sterols concentrations were not associated with an increased risk, but with a reduced risk for CHD [59].

Consumption of plant sterol lead to a total phytosterol concentration which is far below levels seen in homozygous sitosterolemics, who develop premature atherosclerosis [55, 60]. We thus did not discourage phytosterols use and we highlight that our study did not allow to clarify the relation between serum phytosterols and atherosclerosis.

Blood pressure and CRP lowering effect of our tomato sauce (Table 2 and Additional file 3: Figure S2) require confirmation by additional studies, with the change in blood pressure as the main outcome. Previously [10], the antihypertensive effect have been attributed to the stimulation of nitric oxide production in the endothelium by lycopene. We cannot rule out that seasonal influences or increased awareness of having elevated LDL-C led to unintended changes in participants life style, which may have influenced the blood pressure values. Unfortunately we did not measure CRP in the participants taking yogurt.

In our study, the treatment with tomato sauce was associated with a significant reduction of Lp (a) (assessed only in the HA group), that is a particular LDL particle with an added apolipoprotein (a) attached to the apolipoprotein (b) component of the LDL particle, via a disulphide bridge. Apo (a) has homology with plasminogen and correlates with an increased risk of myocardial infarction [61].

The magnitude of the changes in Lp (a) levels due to dietary interventions is relatively modest and several studies have failed to detect any significant effects with a dietary intervention on Lp (a) concentrations [62–64]. Overall, it has been suggested that substitutions of saturated fat with dietary mono- and polyunsaturated fatty acids, as in the Mediterranean Diet, may be better over protein or carbohydrates in relation to Lp (a) concentrations [65].

Studies on the effect of tomato, or its components, and Lp (a) do not exist. Although Lp (a) was only dosed in the HA subgroup, our results confirm that the tomato sauce under study has positive effects on plasma lipids.

The present study has various limitations that need to be considered. First, our study focused on a biomarker, while the effect on the consequently disease would have to be tested. However, we know that abatement of hypercholesterolemia has a statistically significant association in the reduction of risk of CHD, and might there be other beneficial effects as well. However, the presence of other factors and dietary components, which may have influenced the results, cannot be ruled out. Of course, the cost for clinical studies resulting in a health claim is prohibitive. Second, the results cannot be applied to other populations. We acknowledge that not all substances will have the same effect on all or even a majority of consumers. Third, in our study the low adherence rate was 33% (only in the tomato sauce group). However, this was not surprising. There seemed to be a higher average adherence rate for capsules intake compared to food, drink and other forms of supplementation [66]. Moreover, we did not report serum carotenoids data. Serum carotenoid concentrations were quantified by HPLC in frozen samples but unfortunately carotenoids were undetectable. However, in healthy volunteers, after 4 days of tomato sauce consumption, serum lycopene was similar to a previous study [67, 68].

The strengths of this study are the cross-over design as well as the method: we followed patients to assess adherence to the intervention and, most importantly, reported the adherence rate. Due to the finding of a worse lipid profile in low adherent participants compared to those with the highest adherence, and considering that baseline characteristics of participants in the two groups were similar, our results would seem to be confirmed.

## Conclusions

In summary, the results of the present study demonstrate cholesterol-lowering effects of a novel functional from our tomatoes ripened on-the-vine, similar to statins or nutraceuticals. Further studies with a longer duration of intervention are warranted. In the meantime, the increasing body of knowledge on this issue indicates that functional foods may be a useful adjunct to a healthy diet in the management of common hypercholesterolemia in individuals.

## Supplementary Information

**Additional file 1: Table S1.** Servings of various food categories consumed daily or weekly during the study according with the dietary treatment.

**Additional file 2: Figure S1.** Individual LDL-C Reduction according to treatment.

**Additional file 3: Figure S2.** Individual high sensitive C reactive protein Reduction after Tomato sauce.

**Additional file 4.** Individual Lp(a) change after tomato-sauce intake in high-adherence group.

## Data Availability

The datasets used and/or analysed during the current study are available from the corresponding author on reasonable request.

## Abbreviations

BMI: Body mass index
WC: Waist circumference
HC: Hip circumference
SBP: Systolic blood pressure
DBP: Diastolic blood pressure
TC: Total cholesterol
HDL-C: High density lipoprotein cholesterol
LDL-C: Low density lipoprotein cholesterol
TG: Triglycerides
AST: Aspartate aminotransferase
ALT: Alanine aminotransferase
CRP: C-reactive protein
HA: High adherence
LA: Low adherence
MALDI-TOF: Matrix-assisted laser desorption/ionization Time of Flight
