# Supplementary material for: Effect of a novel functional tomato sauce (OsteoCol) from vine-ripened tomatoes on serum lipids in individuals with common hypercholesterolemia: tomato sauce and hypercholesterolemia
Source: J Transl Med. 2021 Jan 6;19:19. doi: 10.1186/s12967-020-02676-3 (PMC7788951; doi:10.1186/s12967-020-02676-3)
Supplement: Supplementary file 1 — Additional file 1: Table S1. Servings of various food categories consumed daily or weekly during the study according with the dietary treatment. [file 12967_2020_2676_MOESM1_ESM.docx]

| Suppl. Table 1*. Servings of various food categories consumed daily or weekly during the study according with the dietary treatment* | | |
| --- | --- | --- |
| Food categories | Tomato sauce | Sterol-enriched yogurt |
| Whole-grain products (serving/d) | 4-5 | 4-5 |
| Fruit/vegetables (serving/d) | 8-10 | 8-10 |
| Nuts (serving/d) | 1-2 | 1-2 |
| Legumes (serving/week) | 2-3 | 2-3 |
| Cheese and yogurt (serving/week) | 2 | 2 |
| Poultry (serving/week) | <3 | <3 |
| Red meat (serving/week) | <2 | <2 |
| Fish (serving/week) | 3-4 | 3-4 |
| Extravirgin olive oil (g/week) | >200 | >200 |
| Sterol-enriched yogurt (serving/d) | / |  |
| OsteoCol (serving/d) | 1 | / |
