# Supplementary figures and images for: Effect of a novel functional tomato sauce (OsteoCol) from vine-ripened tomatoes on serum lipids in individuals with common hypercholesterolemia: tomato sauce and hypercholesterolemia
Source: J Transl Med. 2021 Jan 6;19:19. doi: 10.1186/s12967-020-02676-3 (PMC7788951; doi:10.1186/s12967-020-02676-3)

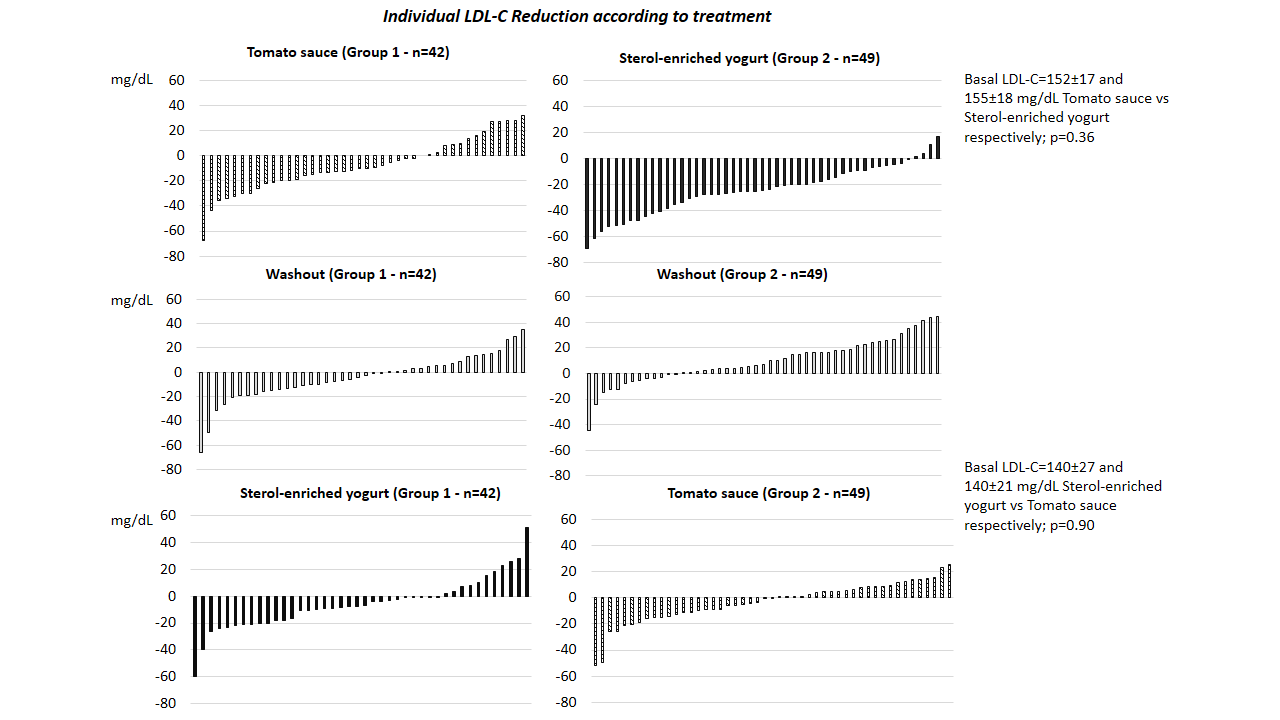

Supplement: Supplementary file 2 — Additional file 2: Figure S1. Individual LDL-C Reduction according to treatment. [file 12967_2020_2676_MOESM2_ESM.tif]

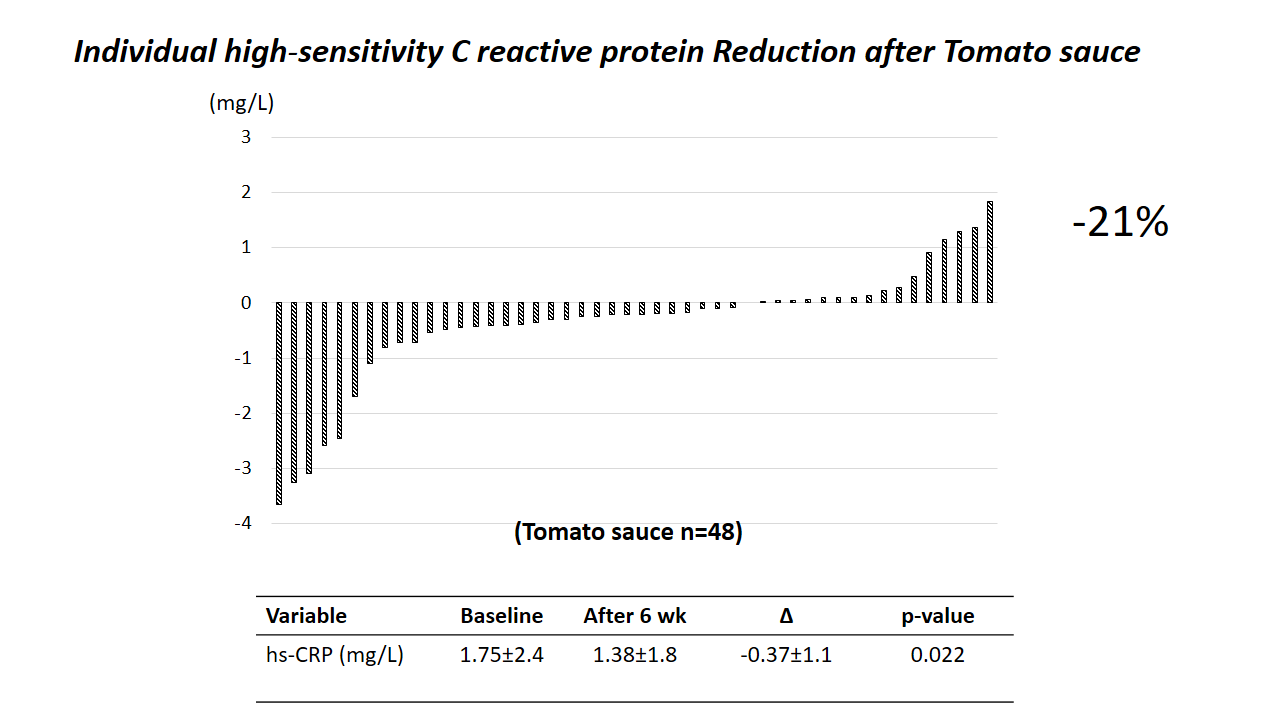

Supplement: Supplementary file 3 — Additional file 3: Figure S2. Individual high sensitive C reactive protein Reduction after Tomato sauce. [file 12967_2020_2676_MOESM3_ESM.tif]

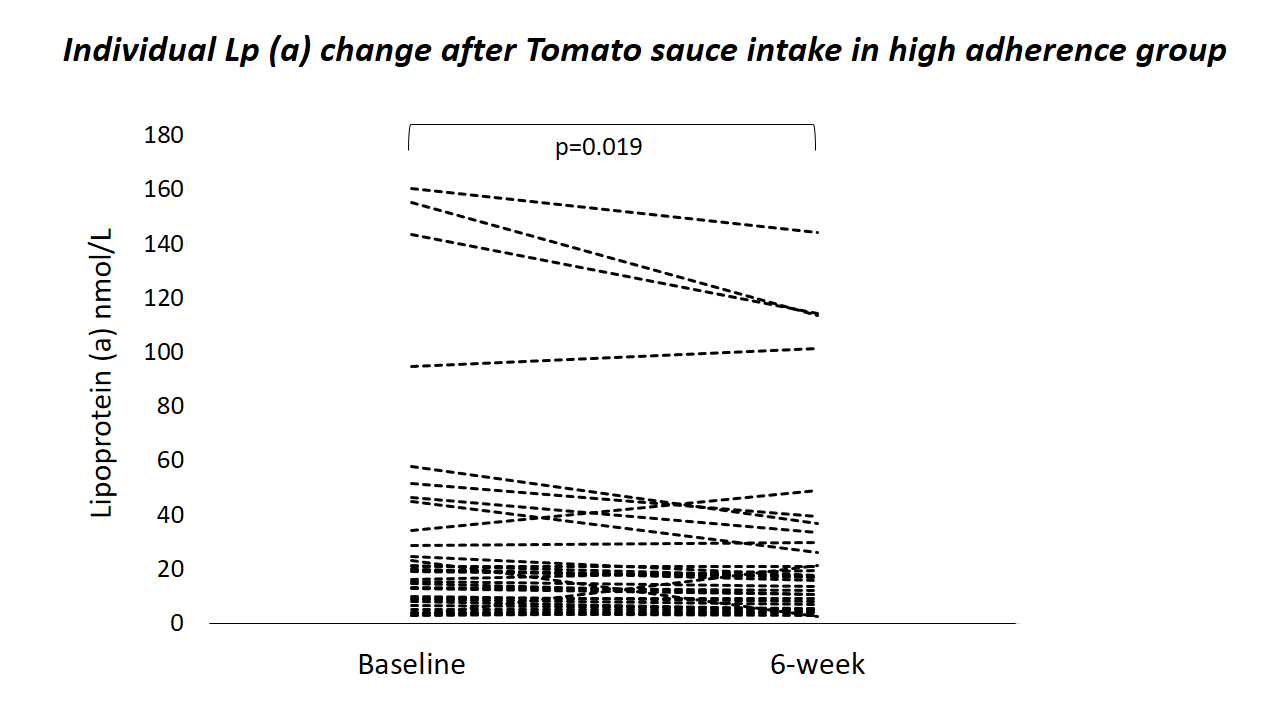

Supplement: Supplementary file 4 — Additional file 4. Individual Lp(a) change after tomato-sauce intake in high-adherence group. [file 12967_2020_2676_MOESM4_ESM.tif]
